# 5-aza-2′,2′-Difluroro Deoxycytidine (NUC013): A Novel Nucleoside DNA Methyl Transferase Inhibitor and Ribonucleotide Reductase Inhibitor for the Treatment of Cancer

**DOI:** 10.3390/ph10030065

**Published:** 2017-07-20

**Authors:** Richard Daifuku, Zhenbo Hu, Yogen Saunthararajah

**Affiliations:** 1 Epigenetics Pharma, 9270 SE 36th Pl, Mercer Island, WA 98040, USA; 2 Department of Hematology, Affiliated Hospital of Weifang Medical University, Weifang 261000, China; zhenbohux@163.com; 3 Cleveland Clinic Taussig Cancer Institute and Case Comprehensive Cancer Center Cleveland, Cleveland, OH 44011, USA; saunthy@ccf.org

**Keywords:** DNA methyl transferase, ribonucleotide reductase, nucleoside, NUC013, decitabine, 5-azacytidine, gemcitabine, p53

## Abstract

Tumor suppressor genes can be silenced genetically as well as epigenetically. One approach to reversing epigenetic suppression of tumor suppressor genes is to inhibit DNA methyl transferase. 5-aza-2′,2′-diflurorodeoxycytidine (NUC013) is a novel DNA methyl transferase and ribonucleotide reductase inhibitor that is a more potent inhibitor of growth than decitabine in the NCI 60 cancer cell line panel. NUC013 is more active than decitabine against p53-null/mutant cancer cell lines (*p* = 0.027) but is even more so against p53 wild-type (WT) cell lines (*p* = 0.0025). The maximum tolerated dose in mice of NUC013 is greater than 120 mg/kg administered intravenously for three consecutive days a week for three weeks. With this regimen and a dose of 20 mg/kg in a human leukemia HL-60 (p53-null) NCr-nu/nu mouse xenograft model (*n* = 10/group), NUC013 demonstrated a survival benefit (saline median survival (MS) = 26.5 days, NUC013 MS = 32 days and hazard ratio (HR) = 0.26 (*p* = 0.032)). In a colon cancer LoVo (TP53 WT) xenograft, mice treated with decitabine at 5 mg/kg had worse survival than saline controls (decitabine MS = 31 days, saline MS > 60 days and HR = 26.89 (*p* < 0.0001)). At a dose of 20 mg/kg NUC013, mean tumor volume in the LoVo xenografts was lower than controls by 50.9% and at 40 mg/kg by 53.7% (both *p* < 0.0001).

## 1. Introduction

An aberrant gain of DNA methylation at the promoter CpG islands and enhancer regions of critical tumor suppressors is observed in most cancers and leads to the inactivation of the expression of genes that suppress tumorigenesis [1]. The genes involved include those that suppress apoptosis, differentiation, metastasis and angiogenesis, those that repair DNA and those that express tumor-associated antigens [2].

**Figure 1:**
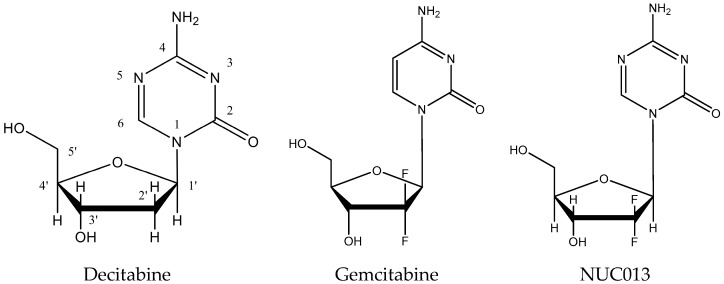
Comparison of the structures of decitabine, gemcitabine and NUC013.

5-aza-2′,2′-difluroro deoxycytidine (NUC013) is a novel nucleoside analog designed to be a DNA methyl transferase inhibitor (DNMTI) and ribonucleotide reductase inhibitor (RNRI). NUC013 consists of the base of 5-azacytidine (Vidaza^®^, Celgene, Summit, NJ, USA) or 5-aza-2′-deoxycytidine, also known as decitabine, (Dacogen^®^, Otsuka America Pharmaceutical, Rockville, MD, USA) conjugated with the sugar moiety of gemcitabine (Gemzar^®^, Eli Lilly, Indianapolis, IN, USA) (see Figure 1).

Both 5-azacytidine and decitabine are approved in the United States for the treatment of myelodysplastic syndrome (MDS). More recently, decitabine has been approved in the European Union for the treatment of acute myeloid leukemia [3]. These nucleosides were first synthesized in 1964 [4,5]. The lack of approved novel molecules in the interim attests to the difficulty in designing safe and effective molecules of this class.

To be active as a DNMTI, 5-azacytidine must first be reduced in vivo to the deoxy-analog, decitabine. Decitabine must then be phosphorylated to decitabine triphosphate and incorporated in DNA before inhibiting DNA methylation [6]. Both drugs produce remissions or clinical improvements in more than half of the treated patients with MDS. Optimization of therapy has included (1) reducing the dose to favor hypomethylation over cytotoxicity; (2) prolonging administration schedules and (3) increasing dose intensity without reaching cytotoxicity. Considering that both have the common side effect of myelosuppression that limits dosing and duration of treatment, it is clear that better inhibitors of DNA methylation are needed for clinical use, particularly ones that have less cytotoxicity and more activity against solid tumors [7,8]. Data from the two currently approved drugs suggest that myeloid malignancies are the neoplasms most sensitive to inhibitors of DNA methylation; however, there is no known reason why solid tumors should not respond as well [9,10].

Gemcitabine is approved as a first line treatment for pancreatic cancer and as combination therapy for other solid tumors. It is an inhibitor of DNA synthesis and, most relevant to NUC013, gemcitabine diphosphate inhibits RNR. Inhibition of RNR causes a reduction in the concentrations of deoxynucleotides, including deoxycytidine triphosphate (dCTP). As gemcitabine triphosphate competes with dCTP for incorporation into DNA, the reduction in the intracellular concentration of dCTP enhances the incorporation of gemcitabine triphosphate into DNA (a mechanism that has been described as self-potentiation) [11].

## 2. Results

### 2.1. NUC013 DNMT Inhibition

NUC013 and 5-azacytidine were first tested for growth inhibition against human colon cancer cell line HCT-116 (TP53 WT), which is also part of NCI 60 cell line panel. The concentration of drug that inhibited 50% of cell growth (GI_50_) for 5-azacytine was 0.62 µM versus 4.54 µM for NUC013. In HCT-116, NUC013 at 1 µM achieves 53.4% inhibition of total DNMT in a colorimetric assay compared to 19.2% for 5-azacytidine (means of 3 experiments, *p* = 0.060, paired *t*-test, two-tailed). At 10 µM, NUC013 inhibition of total DMNT was 48.5% versus 48.6% for 5-azacytidine.

**Figure 2:**
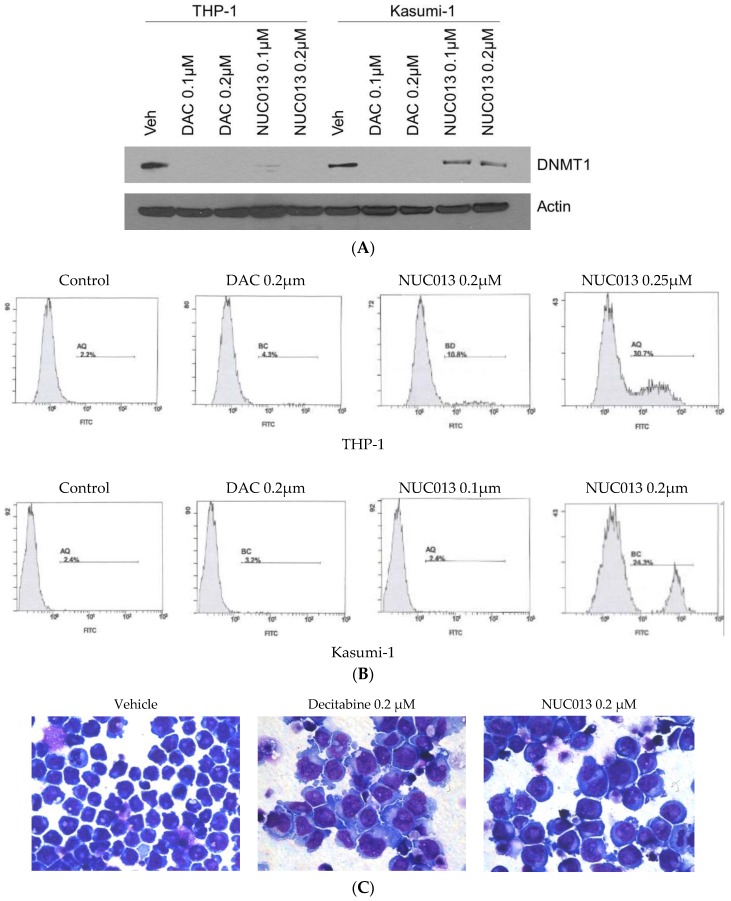
(**A**) Western blot comparing vehicle (veh) to decitabine (DAC) and NUC013 6 days after exposure to 0.1 µM and 0.2 µM of nucleoside and probing with DNA methyl transferase (DNMT1) antibody in human leukemia cells lines THP-1 and Kasumi-1; (**B**) Comparison of induction of apoptosis in THP-1 or Kasumi-1 cells by vehicle control, decitabine (0.2 µM) or NUC013 (concentrations as indicated in figure). Cells were co-stained with Annexin-V and PI on day 2 and analyzed by flow cytometry. Only Annexin-V data are presented because there were no changes in propidium iodide (PI) staining over the time period; (**C**) Photomicrographs of THP-1 cells treated with vehicle, decitabine or NUC013 on day 6 following drug exposure and stained with Giemsa (×630).

These results were confirmed and extended in human leukemia Kasumi-1 (TP53 R248Q mutant) and THP-1 (TP53 null) cell lines. In these cell lines, NUC013 demonstrated DNMT1 inhibition by western blot comparable to decitabine at 0.1 and 0.2 µM in THP-1 cells, though DNMT1 inhibition in the same concentration range is weaker than decitabine in Kasumi-1 cells (See Figure 2A).

In these same cell lines, NUC013 demonstrated apoptosis after 24 h starting at a concentration of 0.25 µM in Kasumi-1 and 0.2 µM in THP-1 (Figure 2B), compared to 0.75–1 µM for decitabine (data not shown). Similar to decitabine, NUC013 resulted in morphologic changes suggestive of cell differentiation 6 days following exposure to study drug (Figure 2C).

### 2.2. NUC013 RNR Inhibition

RNR inhibition by NUC013 was evaluated by exposing Hela cells to drug and then determining by high-performance liquid chromatography (HPLC) whether deoxynucleotide synthesis was inhibited when compared to negative control cells. Gemcitabine was used as a positive control.

Table 1 shows that neither gemcitabine nor NUC013 affected concentrations of ribonucleotides. However, lower concentrations of deoxyribonucleotides are noted from lysates from cells treated with gemcitabine or NUC013, in particular of dATP, dGDP and dUTP. dTDP appears to be unaffected by the presence of NUC013, while the increase in levels of dTDP with gemcitabine may be due to co-elution with gemcitabine-DP.

### 2.3. NUC013 Cancer Cell Line Activity

Extensive testing has been performed on the activity of NUC013 in cancer cell lines, including the NCI 60 cell line panel. The panel comprises human cancer cell lines representing nine different tissues of origin: breast, colon, central nervous system, renal, lung, melanoma, ovarian, prostate and hematogenous. These cells lines are very well characterized, and their TP53 status has been reported in the literature [12].

As of early 2007, all compounds submitted to the NCI 60 Cell Panel have been tested initially at a single dose (10 µM). Hence, it is only possible to compare activity at 10 µM; for decitabine, whether the log GI_50_ is <−5.0 M or ≥−5.0 M, while for NUC013 whether at 10 µM percent growth is ≥50% or <50%. Only cell lines that were tested for both compounds are presented, as the composition of the panel has undergone some minor changes with time (see Supplementary Table S1).

Using the cut-off of 10 µM, it is possible to summarize and compare the activity of decitabine and NUC013 in the NCI 60 cell line panel and determine what influence, if any, the presence of TP53 WT has on growth inhibition by decitabine or NUC013 in 2 × 2 contingency tables. As shown in Table 2, NUC013 was significantly more effective than decitabine in the NCI 60 Cell Line Panel (*p* = 0.0002).

In Table 3, *p* = 0.66 confirmed a very high probability of a true null hypothesis, i.e., that TP53 status had no effect on decitabine GI_50_. In contradistinction, data in Table 4 demonstrated that for NUC013 there was a significant association (*p* = 0.013) between TP53 status and cell growth inhibition. Another way to look at the data is that while a comparison of NUC013 efficacy versus decitabine in TP53 null/mutant cell lines was statistically significant (*p* = 0.027, Fisher’s exact test, two-tailed), results in TP53 WT cell lines reach an even more stringent level of statistical significance (*p* = 0.0025, Fisher’s exact test, two-tailed).

Further testing was performed in colon cancer cell lines. NUC013 was tested against decitabine in two TP53 WT colon cancer cell lines which are not part of the NCI 60 cell line panel: Ls174T and LoVo. The GI_50_ was greater than 50 µM for decitabine in both cell lines, while for NUC013, the GI_50_ was 1.3 µM for Ls174T and 3.0 µM for LoVo (see Supplementary Figure S1).

### 2.4. NUC013 Pharmacology

Pharmacokinetic experiments of intravenous injections of NUC013 in mice demonstrated a half-life of 20.1 min (see Table 5). NUC013 could not be identified at the 6 and 24 h timepoints (see Figure S2).

### 2.5. NUC013 Tolerability

NUC013 was tested in mice at repeat doses of up to 120 mg/kg IV for 3 consecutive days per week for 3 weeks, without evidence of weight loss or death over the time period of observation.

### 2.6. Xenograft Studies with NUC013

NUC013 was tested against a positive control (decitabine) and a negative control (saline) in two mouse models (*n* = 10 per group). Drugs were administered IV for 3 consecutive days a week for 3 weeks. HL-60 is a human leukemia cell line (TP53 null) that is part of the NCI 60 cell line panel and can be grown in nude mice. LoVo is a colon cancer cell line (TP53 WT) that demonstrated good in vitro activity of NUC013.

### 2.7. HL-60 Xenograft Model

**Figure 3:**
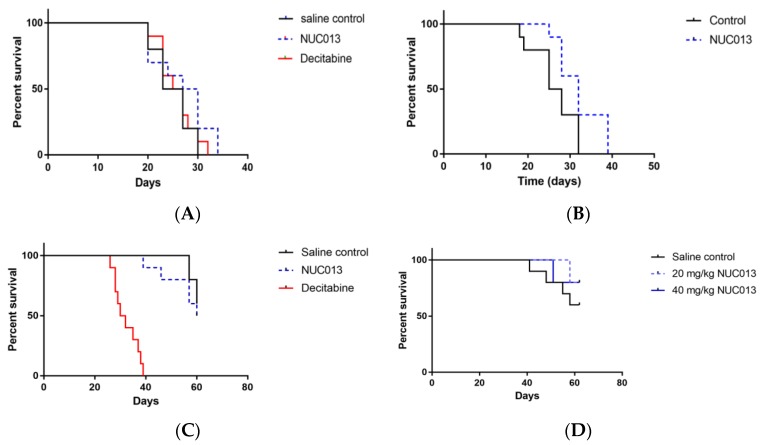
Survival proportions in HL-60 and LoVo xenografts treated with decitabine or NUC013 (*n* = 10/group). “Survival” refers to animals that were not removed from the study by death or per protocol euthanasia. (**A**) Survival proportion in HL-60 xenografts. Saline control compared to decitabine (5 mg/kg) and NUC013 (5.8 mg/kg). Saline median survival (MS) = 25 days, decitabine MS = 26 days, and hazard ratio (HR) = 0.92 (*p* = 0.64, Log-rank test); NUC013 MS = 28.5 days and HR = 0.59 (*p* = 0.19, Log-rank test); (**B**) Survival proportion in HL-60 xenografts. Saline compared to NUC013 (20 mg/kg). Saline MS = 26.5 days, NUC013 MS = 32 days and HR = 0.26 (*p* = 0.032, Log-rank test); (**C**) Survival proportion in LoVo xenografts. Saline control compared to decitabine (5 mg/kg) and NUC013 (5.8 mg/kg). Saline MS undefined, decitabine MS = 31 days and HR of 26.89 (*p* < 0.0001, Log-rank test); NUC013 MS = 60 days and HR = 1.64 (*p* = 0.48); (**D**) Survival proportion in LoVo xenografts. Saline control compared to 20 mg/kg and 40 mg/kg NUC013. MS undefined.

**Figure 4:**
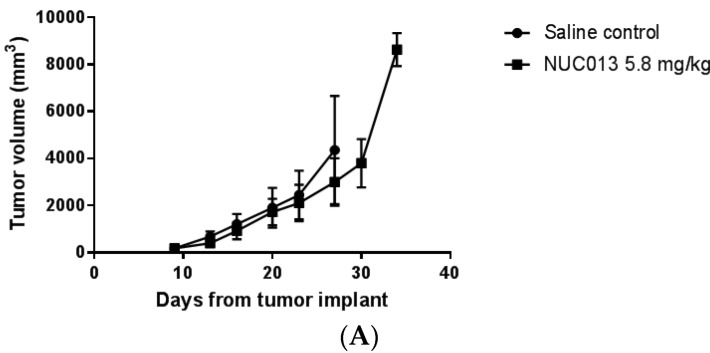

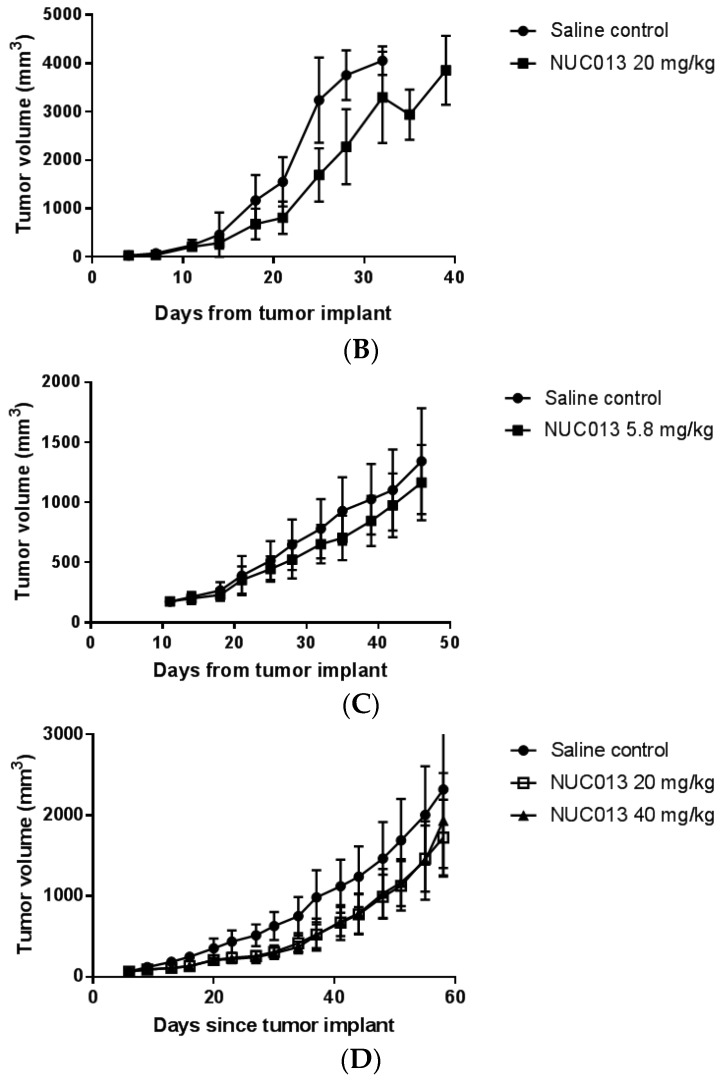
Tumor volume as a function of time since tumor implant. The first data point on each graph is on the day of study drug treatment initiation. Values are ± standard deviation. (**A**) HL-60 comparison of NUC013 5.8 mg/kg vs. saline control; (**B**) HL-60 comparison of NUC013 20 mg/kg vs. saline control; (**C**) LoVo comparison of NUC013 5.8 mg/kg vs. saline control; (**D**) LoVo comparison of NUC013 20 mg/kg and 40 mg/kg vs. saline control.

For HL-60, it is possible to analyze survival because the tumor is rapidly lethal. In the first study, mean tumor volume at treatment initiation was 188 mm^3^. At a dose of 5 mg/kg of decitabine and the equimolar dose of NUC013 to saline control, the median survival (MS) of treated groups did not significantly differ from that of saline control (Figure 3A), nor did tumor volumes (Figure 4A). It is noteworthy though that 4 out of 10 mice died or were euthanized for moribundity in the decitabine group, while none were in the NUC013 or saline groups.

In the second study, the mean tumor volume at study initiation was 33 mm^3^ and treatment with NUC013 (20 mg/kg) significantly improved MS (*p* = 0.032) (Figure 3B). Furthermore, significant decreases in tumor volume (*p* < 0.05, Mann-Whitney U test, two-tailed) were noted on study days 18 through 28 (see Figure 4B), reaching a maximum on study days 21 and 25 (47.8% lower than saline control).

### 2.8. LoVo Xenograft Model

In the first study, treatment was initiated in mice when tumor reached a mean of 173 mg. The data were most striking for the presence of decitabine toxicity at a dose of 5 mg/kg (8 mice died while 2 were euthanized for ulcerated tumors) versus saline (*p* < 0.0001) (see Figure 3C). At equimolar doses of NUC013 8 days following the end of treatment (study day 27), mean tumor volume was lower by 24.3% compared to controls (*p* = 0.048, *t*-test, two-tailed) (see Figure 4C). In the second study, at a dose of 20 mg/kg of NUC013 (mean tumor volume 66 mm^3^ at treatment initiation), the graph shows trends towards improved survival (see Figure 3D). Mean tumor volumes were significantly lower in both treated groups (*p* < 0.05, Mann-Whitney U test, two-tailed) on study days 9 through 51, compared to saline control. As with the dose of 5 mg/kg, mean tumor volume in treated mice decreased the most compared to control mice 8 days following the end of treatment (study day 30): 50.9% in mice treated with 20 mg/kg and 53.7% when mice where treated with 40 mg/kg (both *p* < 0.0001, *t*-test, two-tailed) (see Figure 4D).

## 3. Discussion

In colon cancer cells (HCT-116, TP53 WT), NUC013 showed a greater than 50% total DNMT inhibition at 1 µM. NUC013 achieved this level of DNMT inhibition at a concentration that was lower than its GI_50_ (4.54 µM), while such was not the case for 5-azacytidine (GI_50_ = 0.62 µM) where the concentration required to achieve similar levels of inhibition was greater than 1 µM but likely less than or equal to 10 µM. NUC013 was confirmed as a DNMT1 inhibitor in leukemic cell lines THP-1 and Kasumi-1.

Like all 5-azacytidines, the 5-azacytosine base of NUC013 is susceptible to hydrolysis at the 6-position of the cytosine ring (see Figure 1) with release of a formyl group (data not shown). This susceptibility to hydrolysis is intrinsic to the ability of 5-azacytidines to donate this formyl group to the active serine site of a DNMT resulting in the enzyme’s inactivation [13].

Many 2′-substituted-2′-deoxynucleotides have been shown to be a potent mechanism based RNRI. Detailed studies on 2-fluoro, 2-chloro and 2-azido derivatives have provided the basis for a general mechanism of inhibition by these substrate analogs [14]. There are a number of approaches to determining whether a compound inhibits RNR. One approach is to synthesize a nucleoside diphosphate and then determine whether it inhibits purified RNR or p53R2 [15]. This approach would be technically very difficult with NUC013 because the diphosphate is likely to be quite unstable based on data generated with 5-azacytidine [16] and our experience synthesizing decitabine-MP. The other approach is to expose cells in tissue culture to the drug and determine whether deoxynucleotide synthesis from RNR and p53R2 is inhibited when compared to control cells. Measurement of deoxynucleotides may be done by radioimmunoassay or by HPLC. The disadvantage of a radioimmunoassay is that it can only measure a single deoxynucleotide triphosphate (dNTP) at a time and typically requires a tritiated nucleotide, but it is the preferred method for measuring dCTP [17]. On the other hand, HPLC can measure multiple nucleotides without requiring radiolabeling but has been noted to quantitate poorly dC nucleotides, possibly as an artifact of an influx of dCTP from the medium [18]. In the HPLC assay, both gemcitabine and NUC013 show lower concentrations than control of dATP, dGDP and dUTP. In this assay, inhibition of dATP and dUDP/dUTP synthesis are the hallmarks of gemcitabine inhibition of RNR [19]. dTDP levels are elevated in the case of gemcitabine or unaffected in the case of NUC013. The increased levels in the case of gemcitabine may be related to co-elution of gemcitabine-DP and dTDP and the unchanged levels for NUC013 may be due to the existence of a salvage pathway for formation of the deoxynucleotide independent from RNR [20]. It should be noted that assessment of the relative effectiveness of the compounds as RNRI may vary depending on the cell line used for the assay and that HeLa cells are TP53 null.

The p53R2 gene encodes a small subunit of RNR and has been identified as a p53-inducible gene. While gemcitabine does not induce p53R2, it has been shown to result in its inhibition [15]. Conversely, p53R2 has been shown to be inducible by decitabine and among other activities, p53R2 provides deoxynucleotides in response to p53 activation [21]. More recently, expression of p53R2 has been associated with cancer progression and resistance to therapy [22,23,24]. RNR is a complex between two proteins: the large catalytic protein R1 that contains the allosteric sites and the smaller protein R2. Both proteins are transcriptionally activated during early S-phase and are present in roughly equal amounts to deliver the dNTP required for DNA replication. R2 is degraded during late mitosis and thus postmitotic quiescent cells are essentially devoid of R2 but retain some R1. In postmitotic resting cells, only p53R2 can act as the functioning small subunit of RNR because it is not degraded in mitosis. After DNA damage, p53R2 is transcriptionally activated by p53 and is translocated into the nucleus but has also been shown to be present in the cytosol [25].

Decitabine has been thought of as an S-phase specific agent [26]. However, it has recently been shown that the cell cycle dependence of decitabine is not absolute. Its secondary, epigenetic effects are replication dependent, but its primary effect, DNMT1 enzyme depletion, occurs immediately with elimination half-lives that are unrelated to the fraction of cells in S-phase. Thus, DNA repair and remodeling activity in arrested, confluent cells may be sufficient to support the primary molecular action of decitabine, while its secondary, epigenetic effects require cell cycle progression through S-phase [27]. The ability of NUC013 to inhibit DNMT at a concentration below that causing substantial cell growth arrest favors the expression of its epigenetic effects.

The inhibition of RNR by gemcitabine has been reported to lead to depletion of dCTP, a potent feedback inhibitor of deoxycytidine kinase, leading to a more efficient phosphorylation of gemcitabine. Moreover, since gemcitabine competes with dCTP, a decrease in dCTP pools increases incorporation of gemcitabine into DNA, a mechanism that has been described as self-potentiation [28]. This same mechanism of action is hypothesized to apply to NUC013 and inhibition of RNR and p53R2. Thus, NUC013 can inhibit dNTP synthesis not only during S phase but throughout the cell cycle. In contradistinction, restoration of p53 WT function by decitabine [29] and induction of p53R2 should increase the pool of dCTP, thereby decreasing the incorporation of decitabine during the resting phase, a mechanism that might be described as “self-antagonism”. This self-antagonism may be one reason for the lesser activity of decitabine when compared to NUC013.

Evidence suggests that tumor suppressor gene silencing is an early initiating event in the oncogenic process and that maintaining expression of these genes may well impede the onset of tumorigenesis and tumor progression [1]. The tumor suppressor gene TP53 is of vital importance in preventing human cancer development and progression, and is often referred to as the “guardian of the genome.” Mutations of its gene are detected in approximately 50% of all types of human cancers, and the functions and stability of the p53 protein are often abrogated via posttranslational mechanisms, such as DNA methylation, in the rest of human cancers that harbor TP53 WT. p53 is often inactivated in cancer because it can trigger cell growth arrest, apoptosis, utophagy or senescence, which are detrimental to cancer cells, and it impedes cell migration, metabolism or angiogenesis, which are favorable to cancer cell progression and metastasis [30].

NUC013 has been shown to inhibit the growth of hematogenous tumor cell lines by more than 50% at 10 µM and at least one cell line from all solid tumor tissues tested in the NCI 60 cell line panel, including breast, colon, central nervous system, renal, lung, melanoma, ovarian and prostate. While decitabine is no more effective against TP53 WT than TP53 null/mutant cell lines, NUC013 is significantly more effective (*p* = 0.013) against TP53 WT cell lines. This result is compatible with the experimental data generated by Nieto and others that decitabine unexpectedly induced more apoptosis in TP53 null cells than in TP53 WT cells [31,32,33]. As with decitabine, treatment with NUC013 has also been shown to result in morphologic changes suggesting cell differentiation and to induce apoptosis by a p53 independent mechanism at submicromolar doses. Decitabine has been shown to induce apoptosis in p53 null cell lines by generating reactive oxygen species [34].

Pharmacokinetic experiments of intravenous injections of NUC013 in mice demonstrate a half-life of 20.5 min compared to 30 min reported for decitabine [35]. Cytidine deaminase is considered to be responsible for the relatively short half-life of deoxycytidine analogs in vivo [36,37].

On a schedule of 3 consecutive daily IV doses per week for 3 weeks, NUC013 has been shown to be safer than decitabine in mice. The maximum tolerated dose (MTD) of decitabine is under 5 mg/kg while that of NUC013 is over 120 mg/kg when administered to nude mice on the same schedule, a greater than 24-fold difference. A mean GI_50_ is provided for decitabine in the NCI 60 cell line panel but one is not available for NUC013. However, as a result of GI_50_’s generated for cell lines, such as HCT-116, which were also part of the NCI 60 panel (data not shown), the mean GI_50_ of NC013 can be estimated to be no better than 10-fold lower than decitabine. Hence, the therapeutic index of NUC013 may be conservatively estimated, depending on the actual MTDs, to be in the range of 100- to 150-fold better than decitabine.

In xenograft models implanted with cell lines sensitive to NUC013 and relatively resistant to decitabine, better activity was demonstrated for NUC013. In mice treated with NUC013, survival was significantly improved for a hematogenous tumor (p53 null) and tumor growth significantly inhibited for a solid tumor (p53 WT) compared to untreated mice. This suggests that in vitro activity in other cell lines, whether solid or hematogenous, p53 null or WT, may also translate to activity in vivo.

## 4. Materials and Methods

### 4.1. NUC013

NUC013 was originally synthesized at Sonus Pharmaceuticals (Bothell, WA, USA) and more recently by NuChem Therapeutics (Montreal, QC, Canada).

### 4.2. Total DNMT Inhibition

Total DNMT activity was measured in the colon cancer cell line HCT-116. The assay was performed using the EpiQuick^TM^ DNA Methyltransferase Activity/Inhibition Assay Kit (Epigentek, Brooklyn, NY, USA) [38]. The HCT-116 cells were incubated with 1 µM and 10 µM concentrations of 5-acytidine or NUC013 for a period of 24 h. Assays were performed three times in triplicate.

### 4.3. Western Blot for DNMT1

Approximately 100 µg of cytoplasmic and nuclear protein extracts from cells, together with molecular weight markers, were subjected to SDS-PAGE on 4–12% gradient gels (Invitrogen, Carlsbad, CA, USA) followed by transfer to polyvinylidene fluoride membranes (Invitrogen). Blots were probed using antibodies for DNMT1 (Abcam ab16632).

### 4.4. Apoptosis Assay

Apoptosis was detected by Annexin-V and propidium iodide (PI) co-staining using the APOAF commercial kit (Sigma, St. Louis, MO, USA). Cells (5 × 10^5^) were washed and incubated for 30 min with FITC-conjugated Annexin-V at room temperature. Cells were then resuspended in binding buffer containing PI and immediately analyzed by flow cytometry.

### 4.5. RNR Inhibition Assay

The method used to extract and quantitate nucleotides followed previously reported procedures [39]. Prior to extraction, HeLa cells were treated with 50 µM gemcitabine or NUC013 for 16 h.

### 4.6. NCI 60 Cell Line Panel

Details of the NCI 60 One Dose Screen are well known and available online [40].

### 4.7. In Vitro Activity

Cells were grown in an appropriate medium for the cell line of interest; 96 well plates of each cell line were seeded with 5000 cells per well and left overnight. Drug exposed cells were incubated at 37 °C for 72 h. At the end of the 72 h exposure period, plates were removed for the CellTiter-Glo^®^ assay. Luminescence was recorded on a Synergy 4.0. Assays were performed in triplicate.

### 4.8. Pharmacokinetic Study

This study was carried out in strict accordance with the recommendations of the NIH Guide for the Care and Use of Laboratory Animals. The protocol was approved by the Institutional Animal Care and Use Committee (IACUC) of Eurofin Panlabs Taiwan Ltd (AAALAC Accreditation: 001553).

Twenty-four mice were administered 10 mg/kg NUC013 IV, *n* = 3 animals per timepoint at 8 timepoints (3, 10, 30, 60, 120, 240, 360 and 1440 min) and control animals (*n* = 3, for drug-free blood collection). Blood aliquots (300–400 µL) were collected via cardiac puncture from anesthetized mice in anticoagulant coated tubes, then kept on ice and centrifuged at 2500× *g* for 15 min at 4 °C. The plasma was harvested and kept frozen at −80 °C. Plasma samples were processed using acetonitrile precipitation and analyzed by HPLC-MS/MS. A plasma calibration curve was generated and a reportable linear range determined, along with the lower limit of quantitation.

### 4.9. Tolerability Study

The tolerability study and the tumor xenograft studies (below) were performed at the same institution. These studies were carried out in strict accordance with the recommendations of the NIH Guide for the Care and Use of Laboratory Animals. The protocols were approved by the IACUC of Southern Research Institute (Birmingham, AL, USA) (AAALAC Accreditation: 000643).

Three mice were placed in a dose group on the first day of treatment (Day 1). Animals were treated with the selected dose of NUC013 administered intravenously for 3 consecutive days a week for 3 weeks. Animals were observed daily for mortality and moribundity. Body weights were measured daily starting on day 1. Animals were observed for up to 14 days after the last day of treatment. The MTD was determined by the highest dose at which animals did not die or were not euthanized and had less than 10% weight loss from the start of the study.

### 4.10. Tumor Xenograft Studies

First, 10^7^ tumor cells from culture in Matrigel™ of HL-60 human leukemia or LoVo colon cancer were implanted subcutaneously in the flank of 1.75-fold the number of NCr-*nu/nu* mice required for the study. Study initiation began when the required number of mice had tumors of approximately:

- 100 to 250 mm^3^ (target group mean tumor volume of approximately 175 mm^3^) for the first set of studies.
- 14 to 63 mm^3^ (target group mean tumor weight of approximately 30 mm^3^) for the second set of studies.

Mice with tumors in the proper volume range were arbitrarily assigned to groups. Mice received test article on 3 consecutive days per week for 3 weeks. Mice were observed daily for mortality and moribundity with weights and the tumor measurements taken twice weekly. Tumor volume was determined using the formula for an ellipsoid sphere: Length × Width^2^/2 = Volume (mm^3^). The experiments were scheduled to last for 60 days from the day of tumor implant. Any animal whose weight decreased more than 30% from the weight on the first day of treatment or whose tumor reached 4000 mm^3^ in volume, ulcerated or sloughed off, or was moribund was euthanized prior to study termination.

## 5. Conclusions

NUC013 has been shown to be a DNA methyl transferase inhibitor and an inhibitor of ribonucleotide reductase. In the NCI 60 cell line panel, NUC013 has demonstrated activity against significantly more cell lines than decitabine, particularly against TP53 WT cell lines. The effectiveness of NUC013 against TP53 WT cell lines may be mediated, at least in part, by derepression of TP53 and inhibition of p53R2 thus facilitating incorporation of NUC013 through self-potentiation. In animal models of human leukemia (TP53 null) and human colon cancer (TP53 WT), NUC013 has been shown to be significantly safer and more effective than decitabine at the tested doses and schedules.

## Figures and Tables

**Table 1:** Concentration of nucleotides from HeLa cell extracts comparing treatment with 50 µM gemcitabine or NUC013 vs. control, where A: adenine, G: guanine, T: thymidine, U: uridine, d: deoxy, DP: diphosphate, TP: triphosphate and AU: absorbance units.

Nucleotide	Control, T1	Control, T2	Gemcitabine (50 µM)	NUC013 (50 µM)
**dATP, peak area, AU**	15.8	15.3	0	10.2
**dGDP, peak area, AU**	11.3	10.3	7.95	5.6
**dTDP, peak area, AU**	20.6	18.2	43.4	18.65
**dUTP, peak area, AU**	16.8	35.2	5.3	8.8
**ADP**	14,052	14,188	15,639	13,845
**ATP**	22,163	17,489	26,296	21,335
**GTP**	2003	1151	2036	2006
**CTP**	1209	848	1297	1279
**UTP**	4011	4146	4801	4070

**Table 2:** Allocation of cell lines of NCI 60 cell line panel treated with decitabine or NUC013 by growth inhibition at 10 µM.

Growth Inhibition at 10 µM	Number of Cell Lines	
	Decitabine	NUC013
**>50%**	6	24
**≤50%**	49	31

Fisher’s exact test two-tailed, *p* = 0.0002.

**Table 3:** Allocation of cell lines of NCI 60 cell line panel treated with decitabine by growth inhibition at 10 µM and TP53 status.

Growth Inhibition at 10 µM	Decitabine	
	TP53 (Null/Mutant)	TP53 (WT)
**>50%**	4	2
**≤50%**	36	13

Fisher’s exact test two-tailed, *p* = 0.66.

**Table 4:** Allocation of cell lines in NCI 60 cell line panel treated with NUC013 by growth inhibition at 10 µM and TP53 status.

Growth Inhibition at 10 µM	NUC013	
	TP53 (Null/Mutant)	TP53 (WT)
**>50%**	13	11
**≤50%**	27	4

Fisher’s exact two-tailed, *p* = 0.013.

**Table 5:** Pharmacokinetic parameters of NUC013 after intravenous (IV) administration in mice. Pharmacokinetic parameters were obtained from the non-compartmental analysis of the plasma data using WinNonlin. The analytical method was not validated for NUC013 and did not use addition of tetrahydrouridine, an inhibitor of cytidine deaminase, after specimen collection and, hence, may have resulted in an underestimate of the area under the curve (AUC) and half-life.

Animal	t_1/2_ (h)	C_0_ (ng/mL)	AUClast (h·ng/mL)	AUCInf (h·ng/mL)	AUC Extr (%)	Vss (L/kg)	CL (mL/min/kg)	MRT (h)	Last time point for AUClast (h)
**IV-Mouse**	0.335	36,941	11,082	11,086	0.04	0.26	9.10	0.292	4

## Supplementary Materials

The following are available online at www.mdpi.com/1424-8247/10/3/65/s1, Table S1: Comparison of growth inhibitory activity of decitabine and NUC013 in NCI 60 Cell Line Panel. Activity of NUC013 was only measured at 10 µM. Growth of <50% implies that the GI_50_ is <10^−5^ M and, conversely, growth ≥50% that the GI_50_ is ≥10^−5^ M. Cells in the table were shaded in yellow for GI_50_ ≥10^−5^ M or growth ≥50%, and in green if GI_50_ < 10^−5^ M or growth <50%, Figure S1: (A) Comparison of growth inhibition of p53 WT colon cancer cell line LoVo by decitabine and NUC013. Decitabine GI_50_ > 50 µM and NUC013 = 3.0 µM. (B) Comparison of growth inhibition of p53 WT colon cancer cell line Ls174T by decitabine and NUC013. Decitabine GI_50_ > 50 µM and NUC013 = 1.3 µM, Figure S2: Mean concentration-time profile of NUC013 after IV administration in mice.
