# Supplementary material for: 5-aza-2′,2′-Difluroro Deoxycytidine (NUC013): A Novel Nucleoside DNA Methyl Transferase Inhibitor and Ribonucleotide Reductase Inhibitor for the Treatment of Cancer
Source: Pharmaceuticals (Basel). 2017 Jul 20;10(3):65. doi: 10.3390/ph10030065 (PMC5620609; doi:10.3390/ph10030065)
Supplement: Supplementary file 1 [file pharmaceuticals-10-00065-s001.pdf]

Supplementary Materials: 5-aza-2′,2′-Difluroro Deoxycytidine (NUC013): A Novel Nucleoside DNA Methyl Transferase Inhibitor and Ribonucleotide Reductase Inhibitor for the Treatment of Cancer

Richard Daifuku [1,*], Zhenbo Hu [2] and Yogen Saunthararajah [3]

Table S1. Comparison of growth inhibitory activity of decitabine and NUC013 in NCI 60 Cell Line Panel. Activity of NUC013 was only measured at 10 µM. Growth of < 50% implies that the GI_{50} is < 10^{-5} M and, conversely, growth ≥ 50% that the GI_{50} is ≥ 10^{-5} M. Cells in the table were shaded in yellow for GI_{50} ≥ 10^{-5} M or growth ≥ 50%, and in green if GI_{50} < 10^{-5} M or growth < 50%.

**Panel/Cell line**	**TP53 Status**<br>**(0) Null/mutant**<br>**(1) Wild type**	**Decitabine (-) Log GI_{50} M**	**NUC013**<br>**Growth (%) at 10 µM**
**Leukemia**			
CCRF-CEM	0	4.8	23.67
HL-60	0	4.2	25.23
K-562	0	4.0	65.66
MOLT-4	0	4.2	35.63
RPMI-8226	0	3.9	39.24
SR	1	5.7	14.99
**NSCL**			
A549/ATCC	1	4.6	37.91
EKVX	0	3.8	86.06
HOP-92	0	3.5	34.34
NCI-H226	0	3.7	52.34
NCI-H23	0	3.7	30.61
NCI-H322M	0	3.7	70.00
NCI-H460	1	5.2	9.34
NCI-H522	0	3.9	86.91
**Colon cancer**			
HCC-2998	0	3.8	60.44
HCT-116	1	3.7	29.84
HCT-15	0	5.7	52.19
HT29	0	3.8	70.09
KM12	0	3.7	61.18
SW-620	0	4.0	51.21
**CNS cancer**			
SF-268	0	3.6	65.39
SF-295	0	3.7	77.93
SF-539	0	3.4	22.25
SNB-19	0	3.4	58.88
SNB-75	0	3.7	64.95

**Melanoma**			
LOX IMVI	1	3.7	34.53
MALME-3M	1	3.7	67.90
M14	0	5.0	15.55
MDA-MB-435	0	5.9	53.04
SK-MEL-2	0	3.9	104.47
SK-MEL-28	0	3.7	78.46
SK-MEL-5	1	3.7	70.84
UACC-257	1	4.4	77.54
UACC-62	1	3.7	47.29
**Ovarian cancer**			
IGROV-1	0	3.7	56.77
OVCAR-3	0	3.7	70.56
OVCAR-4	0	4.0	94.88
OVCAR-5	1	3.6	86.95
OVCAR-8	0	4.6	49.95
NCI/ADR-RES	0	3.5	42.13
SK-OV-3	0	3.7	71.09
**Renal cancer**			
786-0	0	5.0	26.29
A498	1	3.5	38.04
ACHN	1	4.2	7.28
CAKI-1	1	3.5	27.86
RXF 393	0	3.6	44.97
SN12C	0	3.7	51.65
TK-10	0	4.7	91.13
UO-31	1	3.4	39.00
**Prostate cancer**			
PC-3	0	5.0	63.57
DU-145	0	4.8	43.48
**Breast cancer**			
MCF7	1	3.8	20.74
MDA-MB-231	0	4.7	79.98
HS 578T	0	5.6	90.37
BT-549	0	5.2	53.63

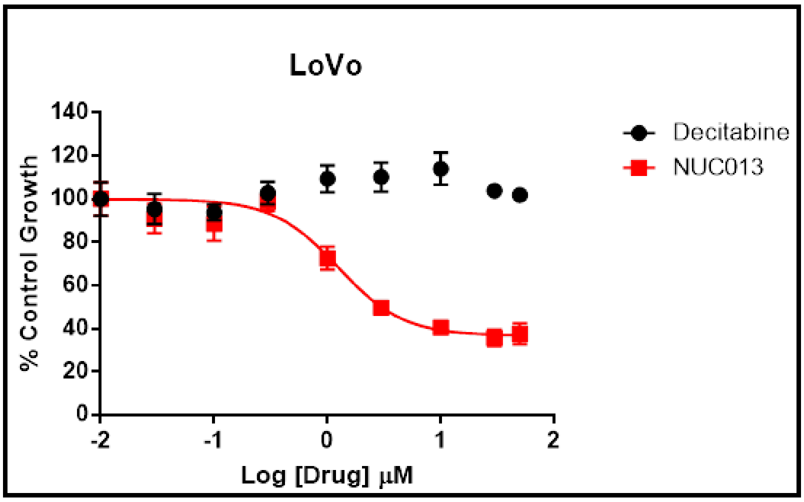

(A)

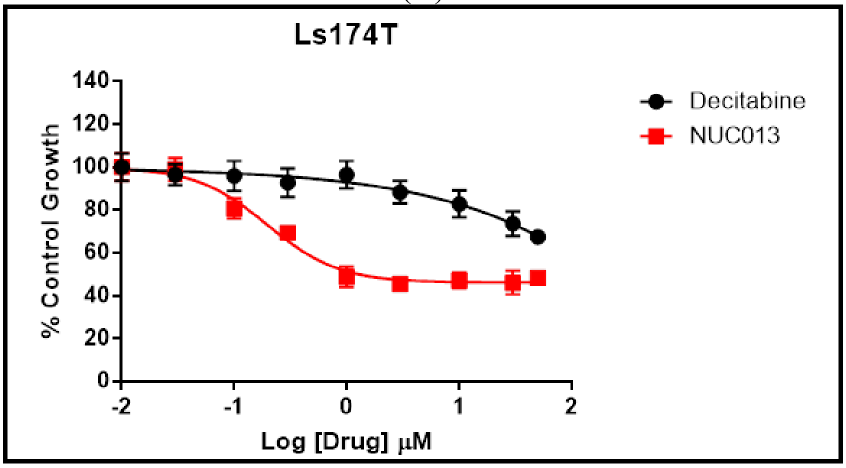

(B)

Figure S1. (**A**) Comparison of growth inhibition of p53 WT colon cancer cell line LoVo by decitabine and NUC013. Decitabine GI_{50} > 50 µM and NUC013 = 3.0 µM. (**B)** Comparison of growth inhibition of p53 WT colon cancer cell line Ls174T by decitabine and NUC013. Decitabine GI_{50} > 50 µM and NUC013 = 1.3 µM.

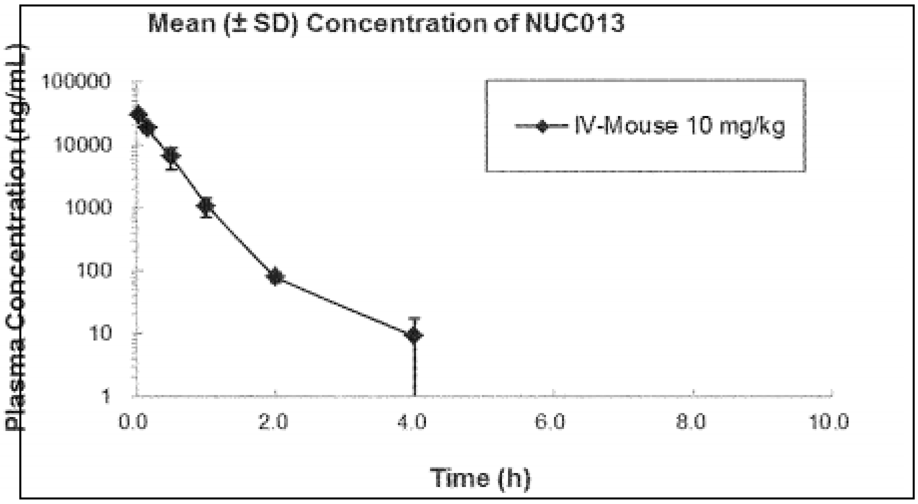

Figure S2. Mean concentration-time profile of NUC013 after IV administration in mice.
